# Accounting for individual differences and timing of events: estimating the effect of treatment on criminal convictions in heroin users

**DOI:** 10.1186/1471-2288-14-68

**Published:** 2014-05-17

**Authors:** Jo Røislien, Thomas Clausen, Jon Michael Gran, Anne Bukten

**Affiliations:** 1 SERAF, Norwegian Centre for Addiction Research, University of Oslo, Kirkeveien 166, 0407 Oslo, Norway; 2 Department of Biostatistics, Institute of Basic Medical Sciences, University of Oslo, Boks 1122 Blindern, 0317 Oslo, Norway

**Keywords:** Maintenance treatment, Criminal activity, Time-to-event, Recurring event, Time-dependent covariate, Dynamic covariate

## Abstract

**Background:**

The reduction of crime is an important outcome of opioid maintenance treatment (OMT). Criminal intensity and treatment regimes vary among OMT patients, but this is rarely adjusted for in statistical analyses, which tend to focus on cohort incidence rates and rate ratios. The purpose of this work was to estimate the relationship between treatment and criminal convictions among OMT patients, adjusting for individual covariate information and timing of events, fitting time-to-event regression models of increasing complexity.

**Methods:**

National criminal records were cross linked with treatment data on 3221 patients starting OMT in Norway 1997–2003. In addition to calculating cohort incidence rates, criminal convictions was modelled as a recurrent event dependent variable, and treatment a time-dependent covariate, in Cox proportional hazards, Aalen’s additive hazards, and semi-parametric additive hazards regression models. Both fixed and dynamic covariates were included.

**Results:**

During OMT, the number of days with criminal convictions for the cohort as a whole was 61% lower than when not in treatment. OMT was associated with reduced number of days with criminal convictions in all time-to-event regression models, but the hazard ratio (95% CI) was strongly attenuated when adjusting for covariates; from 0.40 (0.35, 0.45) in a univariate model to 0.79 (0.72, 0.87) in a fully adjusted model. The hazard was lower for females and decreasing with older age, while increasing with high numbers of criminal convictions prior to application to OMT (all p < 0.001). The strongest predictors were level of criminal activity prior to entering into OMT, and having a recent criminal conviction (both p < 0.001). The effect of several predictors was significantly time-varying with their effects diminishing over time.

**Conclusions:**

Analyzing complex observational data regarding to fixed factors only overlooks important temporal information, and naïve cohort level incidence rates might result in biased estimates of the effect of interventions. Applying time-to-event regression models, properly adjusting for individual covariate information and timing of various events, allows for more precise and reliable effect estimates, as well as painting a more nuanced picture that can aid health care professionals and policy makers.

## Background

Experimental setups and randomized controlled trials have been invaluable to the medical research revolution over the past decades. However, not all diseases and interventions lend themselves to stylized setups, and complex observational data is often the only available source of information. An analytical challenge is the often large heterogeneity between individuals in treatment regimes and the timing of various events. HIV patients drift in and out of treatment [1,2], cancer patients may, or may not, have multiple relapses [3,4] and drug users will change their drug preferences, be enrolled in various treatments, drop out, overdose or die, at varying stages during the course of treatment [5-8]. Nevertheless, accurate and reliable estimates of the effect of often costly interventions are still essential, both for health care professionals and policy makers.

Heroin users as a group have been found to engage in high levels of criminal activity [9-12], and the reduction of crime is an important aspect of maintenance treatment [13]. Several observational studies have found that opioid maintenance treatment (OMT) reduces the level of criminal activity among heroin users [5,14,15]. Estimating of the effect of OMT is however complicated, as OMT patients differ greatly in characteristics and duration of engagement with treatment. Studies have found that patients cycle in and out of treatment, often for multiple episodes [16]. Retention in treatment has consistently been found to be associated with crime outcome [13,17], and longer continuous periods in OMT has been associated with improved outcomes [17].

Individual covariate information and timing of events is however rarely taken into account in OMT research, or when studying criminal activity in heroin users. Focus has been on simple cohort counts and incidence rates, and criminal events grouped based on retrospectively defined criteria conditioned on the termination date of the study [12,18]. This is problematic for several reasons. Firstly, this approach only studies outcomes at a mean group level, not taking intra-individual behavior into account, both with relation to criminal activity and treatment history. Further, without individual based regression models, confounders cannot be properly adjusted for, or associations tested for statistical significance. Finally, not adjusting for censoring introduces bias of unknown direction and magnitude.

Time-to-event analysis, traditionally referred to as survival analysis, is a cornerstone of modern medical statistical analysis, including the seminal work by Cox [19,20]. Over the past decades the field of time-to-event analysis has developed rapidly, and increasingly more complex situations can now be analyzed within the statistical framework of counting processes [21,22]. Approaching the analysis of criminal activity in OMT patients as a set of individual counting processes allows for modeling of individual time courses, with criminal activity as a recurrent event outcome, treatment as a time-dependent covariate, and adjustment for possible confounding variables, both fixed, dynamic, and with time-varying effects.

The purpose of this work was to estimate the relationship between OMT and criminal convictions among heroin users on OMT when adjusting for individual covariate information and timing of events, fitting time-to-event regression models of increasing complexity. We fit univariate and multiple Cox proportional hazards, Aalen’s additive hazards and semi-parametric additive hazards regression models, exploring whether increased model complexity paint a more nuanced picture of the situation than has previously been reported. We find that simple analyses might overestimate the effect of treatment, while including too much detail on the overall process might result in corresponding underestimation.

## Methods

### Data material

Complete records on all 3221 patients who started OMT in Norway from September 1997 through December 2003 were cross linked with official national criminal records from the Norwegian crime statistics (Statistics Norway) December 2007, with information including date of crime and offence details. The last study day was 31 December 2003, thus being the censoring time for all patients, besides the 135 patients who died before this, who were censored at their time of death. The data has been analyzed previously, and a full description on the study settings and participants can be found elsewhere [8,12,18].

The Norwegian crime statistics from 1995 to 2003 provide data on date of all crimes registered by the police in the period, penal code and various prosecution decisions. All convictions are decisions finding a person guilty of a crime in the court of law. In our study, only formal final convictions were included in the analysis. Similarly, convictions on use and possession of illegal drugs were excluded from the analysis, as these data would potentially be particularly influenced by the severity of their drug dependence. We focus here on ‘crime days’; any day with one or more criminal convictions was considered a ‘crime day’.

The patients had from one to six treatment episodes of various lengths. Due to relatively few long observation times, the various time-to-event models were fitted only up until 6 years from application to the OMT programme.

### Ethics

The study was approved by the Regional Committees for Medical and Health Research Ethics, The Norwegian Social Science Data Services (NSD) and the Norwegian Directorate of Health and the Police Directorate. Files were merged and made anonymous by Statistics Norway.

### Statistical methodology

A crude estimate of treatment effect was calculated as the total number of days with criminal convictions in and outside of OMT, divided by the corresponding total number of risk days, that is, the incidence rate. Corresponding confidence intervals were calculated using the non-parametric bootstrap [23].

Following a recent publication on temporal changes in criminal convictions in OMT patients [24] we also calculated the day-by-day mean number of days with criminal convictions in and outside of OMT separately in the following manner. Suppose that *m* individuals are observed over time. Let $m_{t}^{s}$ denote the total number of individuals with treatment status *s* at time *t* and *N*_*i*_(*t*) the number of events at time *t* for the *i*th process. Then the sample mean at time *t* for treatment status *s* under study is ${\hat{\mu }}^{s}\left(t\right)=\frac{1}{m_{t}^{s}}\sum_{m_{t}^{s}}N_{i}\left(t\right)$. A cubic smoothing spline was fitted to this temporal sample mean to ease the interpretation.

We then fitted various time-to-event regression models with increasing model complexity as described below.

#### Counting processes

The flexible mathematical framework of counting processes is specifically designed for handling time-to-event data on an individual level [21,22]. It encompasses statistical techniques like the Kaplan-Meier plot and the log-rank test, as well as Cox proportional hazards and Aalen’s additive hazards multiple regression models, which allow for the adjustment of various types of covariates. In the present study the outcome variable, day with a criminal activity leading to conviction, is a recurrent event. Further, as treatment is not fixed at baseline, but varies over time, it was included as a time-dependent covariate.

#### Fixed covariates

Gender and age at time of application to OMT were added in the regression models as fixed covariates, along with variables describing criminal activity prior to application and criminal activity while on waiting list for OMT. The latter two variables are described below.

Data on criminal convictions from three years prior to application to treatment was included in the material as covariate information, categorized into three categories; patients with no convictions during the pre-treatment period (n = 769), 1–27 convictions (n = 2097), and more than 27 convictions (n = 355).

A natural candidate estimator for the criminal intensity while on waiting list for OMT is the number of days with criminal convictions divided by total number of days while on waiting list. However, as some individuals in the cohort spent zero days on waiting this variable was undefined for these individuals. The variable was also strongly zero-inflated as the majority of individuals had zero crime days while on waiting list. We thus included the dichotomous variable ‘criminal conviction while on waiting list: yes/no’ as a marker for criminal activity while on waiting list for OMT.

#### Dynamic covariates

In order to incorporate the dynamic nature of the problem under study in the analysis, we added two time-dynamic covariates. A dynamic covariate is a type of internal covariate, intermediate between the fixed covariate and the outcome, and may “steal” some of the effect from the fixed covariate, e.g. treatment [22,25]. Using dynamic covariates together with fixed covariates thus requires some caution.

A possibility when modeling the dynamic nature of recurrent events, e.g. criminal convictions, is to include dynamic covariates of the kind ‘time since previous event’ [22]. In our study, however, several individuals had no criminal convictions throughout the observational period, leaving such a variable undefined for several individuals in the cohort. Alternatively, counting the number of events within a recent time window, e.g. last 30 days, also captures some of the dynamics. This variable was well-defined in our data, but was strongly zero-inflated. We thus included the dichotomous variable ‘one or more days with criminal conviction last 30 days: yes/no’ as a dynamic variable in the analyses.

Multiple treatment episodes, as compared to retention in treatment, has been found to be associated with increased criminal activity, and we thus wanted to include the accumulated number of treatments as a dynamic variable in the regression models. However, while individuals had from one through six treatments, relatively few individuals had more than two treatments, and including this as a categorical variable with six ordinal levels led to a breakdown in the model fitting procedures. We thus added as a dynamic variable a dichotomous marker of whether the accumulated number of treatments was more than one.

#### Cox proportional hazards model

We fitted univariate, multiple and dynamic Cox proportional hazards regression models [19,20]. The appropriateness of the Cox model was assessed by plotting scaled Schöenfeld residuals and an accompanying test for zero slope therein [26]. While examining this model diagnostic reveals whether the proportional hazards assumption is met or not, such residual plots will not so easily give information on the nature of non-proportionalities.

#### Aalen’s additive hazards model

A typical deviation from the Cox model is that the effect of covariates change with time; a treatment might have one effect initially, which is later weakened. Two well-known reasons for such diminishing effects are aging measurements and frailty [22]. Aging means that baseline variables become less representative over time, and their effect thus smaller. The concept of frailty, on the other hand, is that, as time goes by, the individuals left in the study are less “frail”, e.g. died or dropped out. Hence, when comparing treatments, the remaining individuals in both groups will be less and less likely to experience an event the higher the initial risk, and the treatment effect will decrease with time. Frailty models aim to account for such unobserved heterogeneity between groups.

Whether a treatment effect is time-varying or constant with time might often itself be the main research question in an analysis. An alternative to the Cox model, which does not condition on constant proportional hazard over time, is Aalen’s additive hazards model [27]. Here covariate effects are allowed to vary freely over time. Rather than the hazard rate, the target for such an analysis is cumulative regression functions. These are plotted against time to give a description of how the covariates influence the outcome over time, and it is the change in the cumulative functions, i.e. the slope, that is of primary interest. Dynamic analysis of recurrent event data using the additive hazards model has been suggested in the statistical literature previously [28].

#### Semi-parametric additive hazards model

A useful sub-class of the additive hazards model is the semi-parametric additive hazards model [29]. Here the effect of some covariates is allowed to vary with time, while the effect of other covariates is assumed to be constant.

#### Calculations

The statistical analysis was performed in R 2.12 [30]. The Cox proportional hazards regression models were fitted using package coxph, based on the counting process formulation of Andersen and Gill [19]. Aalen’s additive hazards and semi-parametric additive hazards regression models were fitted using package timereg, based on the work of Martinussen and Scheike [31]. The effect of the recurrent criminal events and time-dependent treatment covariate on an individual level was controlled for using robust estimation [32]. P-values below 0.05 were considered statistically significant.

## Results

### Incidences

The number of crime days per 1000 days for when in OMT and not was 1.16 and 3.87, respectively, giving an incidence ratio (95% CI) of 0.39 (0.36, 0.41). That is, for the cohort as a whole there is approximately 60% fewer days with criminal convictions when in treatment. Performing subgroup analysis of incidence ratios for men and women separately, we find similar incidence ratios for both groups, with 0.39 (0.36, 0.41) for men and 0.41 (0.36, 0.46) for women. That is, the relationship between treatment and criminal convictions appears to be similar for both genders. Women are generally considerably less criminally active than men, and performing subgroup analysis for in and out of treatment separately, we find incidence rate ratios of criminal convictions for women as compared to men to be 0.50 (0.45, 0.56) for when in treatment and 0.48 (0.43, 0.53) otherwise. That is, men generally have twice as many days with criminal convictions as do women, whether in treatment or not.

**Figure 1:**
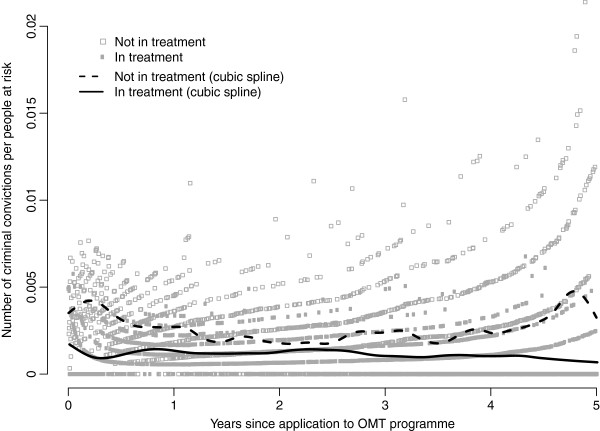
**Day-by-day mean number of criminal convictions.** Day-by-day number of criminal convictions (crime days) per people at risk (grey dots), with cubic smoothing spline superimposed (black lines), for a cohort of 3221 Norwegian heroin users when in opioid maintenance treatment (OMT) and not.

**Figure 2:**
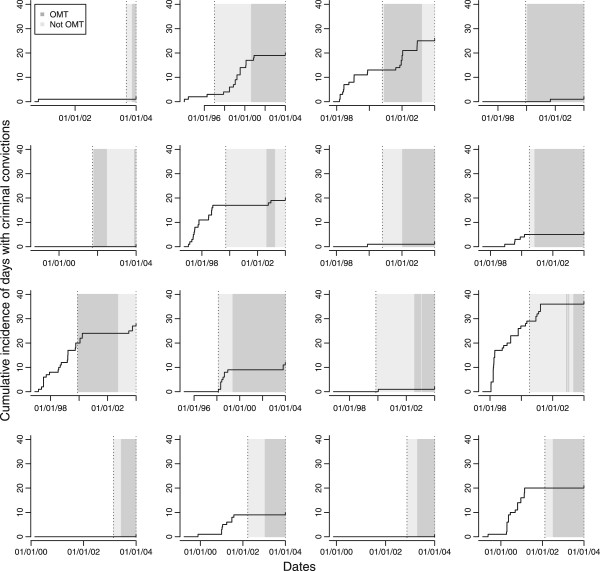
**Individual cumulative incidence of criminal convictions.** Cumulative incidence of criminal convictions (crime days) for 16 random individuals in opioid maintenance treatment (OMT) from application to OMT (left dotted line) to study end December 31^st^ 2003 (right dotted line). Periods not in treatment (light grey area) and in treatment (dark grey area).

The day-by-day number of days with criminal convictions per individuals at risk throughout the observational period appears to be markedly lower when the individuals in the cohort are in treatment as compared to when they are not (Figure 1). The cumulative incidence for 16 random individuals demonstrates large individual differences not accounted for in the crude analyses above (Figure 2).

### Cox proportional hazards model

Results from Cox proportional hazard regression models with crime days as a recurrent event and treatment as a time-dependent explanatory variable is shown in Table 1. We fitted univariate models for all eight covariates and three multiple models with increasing number of covariates. In univariate analysis, being in treatment more than halves the hazard for criminal convictions, HR (95% CI) 0.40 (0.35, 0.45). Older age and being female also reduces the hazard for criminal convictions, while many criminal convictions prior to application, criminal convictions while on waiting list, more than one OMT-episode, and criminal convictions past 30 days all increase the hazard for criminal convictions. All univariate estimates are however attenuated when adjusting for other covariates in multiple regression models. In the full model with all eight covariates, fixed and dynamic, being in treatment is estimated to reduce hazard for criminal convictions by approximately one fifth, HR (95% CI) 0.79 (0.72, 0.87). This is substantially less than in the unadjusted analysis. Results from Cox proportional hazards regression models should however be interpreted with care, as the models fail the test for the assumption of proportional hazards (not shown).

**Table 1:** Cox proportional hazards regression models

	**Univariate regression models**		**Multiple regression model 1**	
**Variable**	**Hazard ratio (95% CI)**	**p-value**	**Hazard ratio (95% CI)**	**p-value**
OMT	0.40 (0.35,0.45)	<0.001	0.79 (0.72,0.87)	<0.001
Female gender	0.48 (0.40,0.57)	<0.001	0.75 (0.66,0.85)	0.001
Age [10 years]	0.60 (0.54,0.67)	<0.001	0.79 (0.73,0.85)	<0.001
>27 criminal days prior to OMT application	4.83 (4.19,5.55)	<0.001	1.50 (1.35,1.66)	<0.001
Criminal conviction while on waiting list	6.65 (5.70,7.76)	<0.001	2.84 (2.47,3.25)	<0.001
>1 OMT period	1.74 (1.33, 2.28)	<0.001	1.43 (1.20,1.70)	<0.001
Criminal conviction last 30 days	93.9 (87.1, 101.2)	<0.001	45.2 (40.4, 50.5)	<0.001

Cox proportional hazards regression models with day of criminal conviction as a recurrent event outcome and opoid maintenance treatment (OMT) a time-dependent explanatory variable. Results should be interpreted with care as the multiple model failed the assumption of proportional hazards.

### Aalen’s additive hazards model

**Figure 3:**
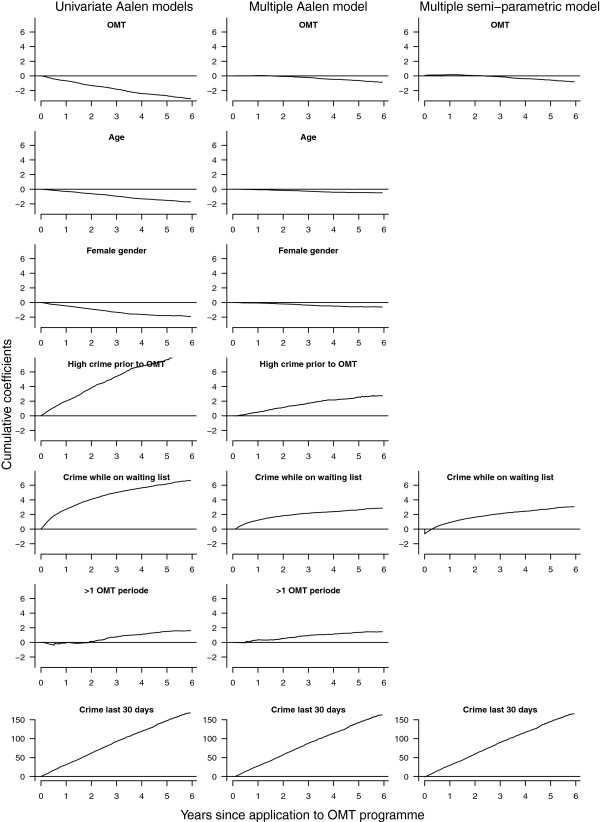
**Additive hazards regression models.** Univariate and multiple Aalen’s additive hazards regression models for days with criminal convictions as a recurrent event dependent variable and treatment as a time-dependent covariate, and the non-parametric components of a multiple semi-parametric additive hazards regression model.

Results from Aalen’s additive hazards regression models with crime days as a recurrent event and treatment as a time-dependent explanatory variable is shown in Figure 3. We fitted univariate models for all eight covariates and a multiple model with all covariates. Accompanying statistical tests show that all variables are highly statistically significant in all models (all p < 0.001). The effects of treatment, criminal convictions while on waiting list and criminal conviction past 30 days are significantly time-varying (all p < 0.05). All univariate estimates are attenuated when adjusting for other covariates in multiple regression models (Figure 3).

### Semi-parametric additive hazards model

Results from Aalen’s additive hazards regression model indicate that the effect of age, gender criminal activity prior to OMT and accumulated number of treatments can be assumed to be constant over time. Results from the corresponding semi-parametric additive hazards regression model are shown in Table 2 and Figure 3. All variables are strongly statistically significant (all p < 0.005). The evidence of time-varying effects of treatment, criminal convictions while on waiting list and criminal conviction past 30 days is strengthened (all p < 0.025).

**Table 2:** Parametric terms of semi-parametric additive hazards model

	**Multiple regression model 2**	
**Variable**	**Hazard ratio (95% CI)**	**p-value**
OMT	(See Figure 3)	<0.001
Female gender	-0.0088 (-0.0142, -0.0034)	0.001
Age [10 years]	-0.0006 (-0.0010, -0.0003)	<0.001
>27 criminal days prior to OMT application	0.0463 (0.0284, 0.0641)	<0.001
Criminal conviction while on waiting list	(See Figure 3)	<0.001
>1 OMT period	0.0212 (0.0080, 0.0343)	0.001
Criminal conviction last 30 days	(See Figure 3)	<0.001

Effect estimates for parametric terms of a semi-parametric additive hazards regression model with day of criminal conviction as a recurrent event outcome and opoid maintenance treatment (OMT) a time-dependent explanatory variable. Time-varying covariate effects are shown in Figure 3.

## Discussion

In the present study we have fitted various time-to-event regression models in order to explore the relationship between opioid maintenance treatment (OMT) and criminal convictions in heroin users. Previous analyses have focused on simple incidence rates and subgroup analyses [18]. We refine these results by replacing simple cohort averages with estimates from regression models in prospective time, adjusting for individual covariate information and timing of events. While these effect estimates not necessarily represent *causal* effects, they are a marked improvement over mere counts. Notably, even the simplest of these time-to-event models might be considered relatively complex in the larger body of the research literature in the field, being the first study of OMT data to include both a recurrent event outcome and a time-dependent treatment variable.

Simple, unadjusted analyses are a natural first step in any data analysis. However, it must still be a first step in the right direction. When analyzing timed events, unless every individual under study has been allowed sufficiently time to experience the event(s) or not, time-to-event data will be censored at a time selected by the analyst. Not accounting for this censoring will result in a bias of unknown direction and magnitude. While the mathematical framework of counting processes automatically adjust for this, other, simpler, approaches do not. Defining categories like in-treatment, between-treatments and after treatment, when people can have an unknown number of treatments and be eligible for more treatments for an unknown amount of time, implies using information from the future; whether a non-treatment period is “in-between” or “after” treatment(s) depends on the end-date of the study, i.e. the censoring date. Applying time-to-event regression models helps to improve the quality of the information extracted from data like this.

In the OMT data under study, the naïve incidence rate ratio estimate indicate about 60% fewer criminal convictions while in treatment as compared to not in treatment, for both men and women. The individual based unadjusted Cox model also shows strong effect of treatment, with being in treatment reducing HR for criminal convictions by more than 50%. However, this result is strongly attenuated in the full model, adjusting for fixed demographic covariates and dynamic covariates. Indeed, in the full model the estimated reduction in HR for criminal conviction when in treatment is reduced to about one third, to approximately 20%. Crude, unadjusted estimates of the effect of OMT should consequently be interpreted with care.

It is worth noting that the reduction of the estimated effect of treatment is not mainly due to adjusting for traditional covariates such as age and gender, but when adjusting for covariates related to the particular problem under study, such as baseline crime, current crime and accumulated number of treatment periods. The two latter are dynamic covariates, and such internal covariates are known to “steal” some of the effect of the fixed covariates, e.g. treatment [22]. If the research goal is the overall effect of treatment, adjusting for previous criminal activity can be misleading, as the result is an estimate of the *direct* effect of treatment, not the *total* effect. If however the effect of the covariate, such as previous criminal activity, is of potential interest per se, such adjustment is causally interesting; adding covariates adds to the understanding of the processes and mechanics of the situation under study. That is, what covariates to adjust for or not depends on the research question, and more complex models should be handled with care.

Note that the outcome measure in the present analysis, day with criminal conviction, is not merely an event, but an activity, setting the analysis apart from the analysis of, say, relapse of tumors. When analyzing things we *do*, that is, events that are partly, or fully, a personal choice, rather than something that merely happens to us, the inclusion of covariates should be given extra thought.

The Cox model is a widely used time-to-event model in medical research. However, while many real survival data meet the assumption of proportional hazards, this assumption does not generally hold. It is well-known in the statistical literature that a common feature in time-to-event studies is that covariates “age”, i.e. their effect weakens over time [22]. A treatment might have an effect initially, but the effect wears off as time passes, or it takes some time before an intervention has effect. Such time-varying effects are not naturally easily discovered in the Cox model, but there are workarounds [22]. The Cox model is also known to have problems with some dynamic covariates [22]. While stratifying is a common way of resolving such issues with the Cox model, there are problems with this approach in the data under study. Firstly, stratifying on a variable makes it impossible to estimate the effect of that variable. As a primary aim in this methodological study was to explore the relationship between treatment and criminal convictions, stratifying on treatment groups was not a preferable alternative. Secondly, with repeated events there might be time-varying effects other than those caused by differences between strata; even *within* strata there might be time-varying covariate effects. In this study related for instance to the fact that treatment can start, and stop, at different times, and for a different number of times for each individual.

Aalen’s additive model easily handles dynamic covariates and covariates with time-varying effects [28], with the latter being immediately apparent in the accompanying plots. Interpreting results is somewhat less straightforward, as it is the gradients in plots of cumulative regression functions that are central, not straightforward tabulated, fixed numbers. Thus, in the absence of time-varying effects, a simpler approach might be preferred. The semi-parametric additive hazards model allows for a mixture, with the effect of some covariates being allowed to vary with time, while the effect of others is assumed to be constant. In our data such a model appears to be the best of both worlds, correctly accounting for the time-varying effects, while strengthening the results from the constant terms. Note that multiple-state-models [33,34] might also be a fruitful alternative for this type of data, along with recent developments within the field of causal inference [35,36].

Estimation of causal effects from observational data has been given a lot of attention in recent years. In his 2010 Armitage lecture Aalen focuses on the value of integrating longitudinal data and survival analysis when trying to understand treatment effects [37], and has also discussed a dynamic viewpoint to causality, mediation and time [38]. The traditional way of unraveling direct and indirect effects, by comparing exposure effects in regression models with and without the mediator, will often produce flawed results, among others due to confounding from time-dependent covariates [39]. More sophisticated methods are thus needed. A counterfactual approach to within-individual causal effects has been taken in an analysis of whether marriage reduces crime [40], studying 500 high-risk boys, incorporating extensive time-varying covariates. The fact that substance related questions, such as the issue of a causal relationship between illegal drug use and selling and violent behavior, still remains unresolved, despite a vast number of empirical studies, has been attributed to methodological weaknesses that prevent causal inference [41]. However, whether a causal model approach can indeed be taken in addiction and crime research is unclear, as both basic and applied research on the relationships among drug use and crime readily illustrates threats to the validity of causal inference, as even the issue of temporal order remains unanswered: Which comes first, *drug use* or *crime*? [42].

In our data including variables on the dynamics of the situation under study helped uncover other important variables than treatment. While refining the estimate of the relationship between treatment and criminal convictions was the main aim of the study, the analysis showed that the strongest predictor by far was criminal convictions past 30 days. That is, while criminal activity is relatively uniformly distributed for the cohort as a whole, as indicated by the rate ratio estimates, with a reduced level while in treatment, on an individual level criminal convictions are clustered. One should consequently be particularly aware of individuals who have recently committed a criminal offence, as these individuals are in the high risk group of committing a new offence – irrespectively of whether they are currently in treatment or not. Clinically many of these individuals might be considered as having an antisocial personality, hence a specific trait that characterizes them both inside and outside of treatment.

The introduction of the simple idea of counting events in treatment and non-treatment groups has been invaluable for the advancement of medical research. However, it is not merely *whether* an event occurs that holds information, but often as much *when* it occurs. Including this additional attribute in the analysis will however often dramatically increase the analytical complexity. Reliable estimates of treatment effects are however still crucial in order to paint a truthful picture of the various associations in the data set under study. Statistical methods for analyzing complex time-to-event data are well-known in the statistical literature, and the flexible framework of counting processes can model situations far more complex than what is common in the medical research literature. Such models are still relatively rare in the medical research literature, despite the necessary computer code being freely available.

## Conclusions

For time-to-event data, be it complex observational drug treatment data as presented here, treatment of chronic diseases or other observational data, regression models based on the framework of counting processes can handle large heterogeneity among individuals, both in exposures, treatment regimes and outcomes, as well as paint a more nuanced picture of the situation by adjusting for fixed and dynamic covariates, and time-varying confounding effects, ultimately providing more precise and reliable information that can aid health care professionals and policy makers.

## Competing interests

The authors declare that they have no competing interest.

## Authors’ contributions

JR performed the statistical analyses and drafted the manuscript. JR, TC, JMG and AB all contributed to the discussions about the topic, revision of the manuscript, and to the final approval of the manuscript.

## Pre-publication history

The pre-publication history for this paper can be accessed here:

http://www.biomedcentral.com/1471-2288/14/68/prepub
